# Increasing Internal Medicine Trainees’ Satisfaction With Their Formal Teaching Program: A Quality Improvement Project During the NHS Workforce Crisis

**DOI:** 10.7759/cureus.71005

**Published:** 2024-10-07

**Authors:** Lennart Marahrens, Adam Twigg

**Affiliations:** 1 Rheumatology, Royal Free London NHS Foundation Trust, London, GBR; 2 Internal Medicine, Royal Free Hospital NHS Foundation Trust, London, GBR

**Keywords:** internal medicine training, junior doctor training, medical education research, quality improvement project, teaching and training residents and medical students

## Abstract

The goal of this quality improvement project was to improve internal medicine trainees' satisfaction with their formal teaching program. Initially, qualitative and quantitative data were collected from trainees, which demonstrated an overall negative attitude towards their teaching. Based on the feedback collected, changes were made to the teaching program, including switching to face-to-face teaching, using study leave in trainees' rosters to allow for higher attendance rates and planning subject-specific "teaching afternoons". After these interventions, trainees' satisfaction with their teaching improved substantially in areas of teaching quality, relevance and reliability. This quality improvement project demonstrates that low-cost interventions based on trainee feedback can improve satisfaction with teaching, which has the potential to alleviate workforce challenges in the UK healthcare system.

## Introduction

Postgraduate education and continued professional development of clinicians form two of the three cornerstones of medical education [1]. It has long been argued that for postgraduate doctors in the United Kingdom, formal medical education has been neglected in favor of simply gathering clinical experience through working long hours with direct involvement in patient care [2]. This may be particularly true and acute following the COVID-19 pandemic, which has had a tremendous impact on the way we train and educate doctors after graduation [3]. Despite this, participation in formal postgraduate education remains a mandatory component of the internal medicine curriculum in the United Kingdom [4], which stipulates a minimum number of teaching hours, as well as themes and specialties that must be covered during teaching. Access to formalized and protected teaching has been identified as a key factor affecting the work force retention crisis among doctors [5]. Regular course evaluation by trainees is an important part of improving education [6]. Therefore, auditing internal medicine trainees’ perception of their teaching and taking their feedback into consideration when designing a local teaching curriculum can improve trainees’ satisfaction with their training overall.

This article was previously presented as a poster at the Royal College of Physicians Virtual Poster Competition, on September 16, 2024.

## Materials and methods

We collected anonymous quantitative and qualitative feedback from 22 internal medicine trainees across three different year groups at a large tertiary hospital (Table 1). Surveys were sent out by email and completed online. Respondents were asked to provide feedback based on the teaching that had taken place over the previous 12 months. Quantitative data was collected on a Likert scale in response to 11 specific statements covering the domains, Relevance, Quality, and Reliability of teaching. Three overall statements with a broader scope were asked at the end along with a space provided for free-text responses to give qualitative feedback. All 14 statements alleged a positive opinion of the course. To allow for a simple numerical comparison of responses to statements between cycles, each response choice for a given question was assigned a score as follows: strongly disagree (-2), disagree (-1), neither agree nor disagree (0), agree (1), and strongly agree (2).

**Table 1:** Survey response rate

Year Group	Baseline Survey	Post-intervention Survey
Internal medicine trainees Y1	80% (8/10)	56% (5/9)
Internal medicine trainees Y2	86% (6/7)	90% (9/10)
Internal medicine trainees Y3	100% (5/5)	40% (2/5)
Total	86% (19/22)	86% (19/22)

Following the first cycle of data collection, significant changes to the curriculum were made that were partly based on the specific feedback we obtained. Specifically, all teaching was changed to be in-person (with the option of viewing it online available), dedicated study leave for teaching was included in trainees' rotas wherever possible, and sessions were changed from weekly one-hour seminars to less frequent, but longer "teaching afternoons". Specialties were approached at the beginning of the academic year and asked to prepare one to two "teaching afternoons" each and given a date for these sessions well in advance. In the second cycle, further feedback was then obtained to assess the effectiveness of our intervention. The questionnaire that was sent out in the second cycle was identical to the one used in the first cycle to allow for comparison. Questions used in the survey can be found in Table 2.

**Table 2:** Questions in the feedback survey sent to internal medicine trainees Questions 1-11 (Q1-Q11) were spread across domains of relevance, quality, and reliability. Three additional questions assessed overall teaching satisfaction. Respondents specified their level of agreement or disagreement using the following Likert items: strongly agree, agree, neither agree nor disagree, disagree, strongly disagree.

Relevance	Quality	Reliability	Overall
Q1: The teaching I have received has been relevant to my learning objectives	Q5: The teaching I have received has improved my clinical practice	Q9: Prior to attending, I am confident that the teaching will go ahead as planned	I have received an appropriate number of teaching sessions over the last year
Q2: The teaching I have received has been of relevance to my day-to-day practice	Q6: The teaching I have received has improved my understanding of the topics covered	Q10: Prior to attending, I am confident the teaching will be on the topic listed on the schedule	The teaching has overall been of an acceptable quality over the last year
Q3: The teaching I have received has been pitched at an appropriate level for me	Q7: The teaching I have received has increased my confidence in managing the topics covered	Q11: Prior to attending, I am confident the teaching will be at the location listed on the schedule	I have been satisfied with the quality and quantity of teaching over the past year
Q4: The teaching I have received has been useful for membership exams	Q8: The standard of teaching received has been appropriate for an Internal Medicine teaching course

## Results

At baseline, approximately half of internal medicine trainees felt that the teaching had not been relevant to their curriculum, day-to-day practice, or membership exams. Similarly, less than half of trainees felt that the teaching improved their understanding of the topics. Over two-thirds of trainees said teaching delivery was unreliable and subject to frequent cancellations. Qualitative feedback showed that trainees were particularly unhappy about the high frequency of online teaching and lack of case-based learning; there were concerns regarding achieving the required teaching hours; suggestions for topics included teaching on specialized tertiary services that the hospital provided.

**Figure 1:**
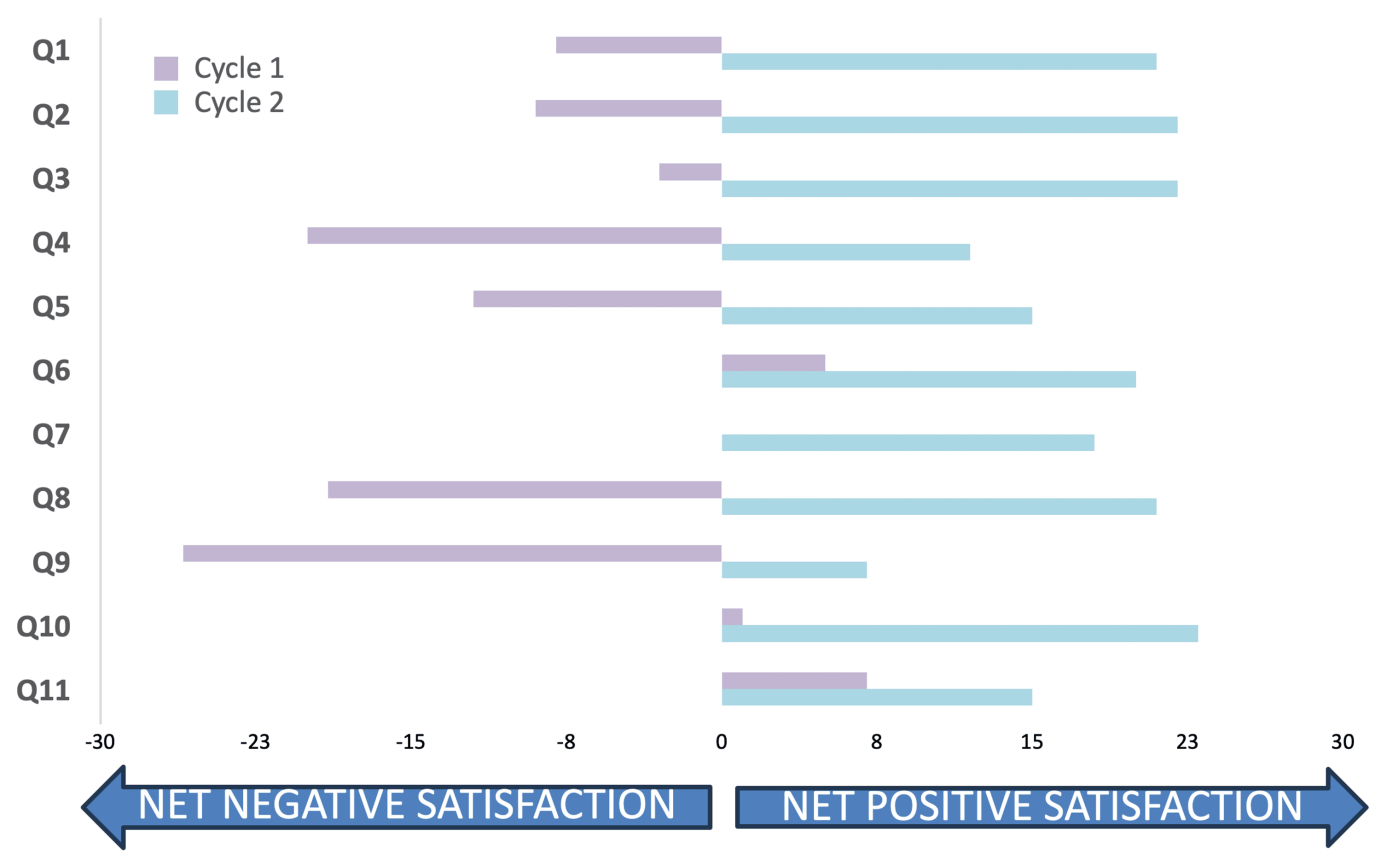
Teaching satisfaction as assessed in 11 questions using Likert scale responses across both cycles Eleven questions (Q1-Q11) were spread across the three domains of Relevance (Q1-4), Quality (Q5-8), and Reliability (Q9-11). See Table 2 for these questions. An overall calculated "agreement score" (X-axis) is shown in response to each of the 11 positive statements put to trainees (Y-axis). Scoring is outlined in the Materials & Methods section. A score greater than 0 indicates overall agreement with the statement, and a score less than 0 indicates disagreement. Cycle 1 (purple) is compared to Cycle 2 (light blue).

In the second cycle, following changes to the teaching, internal medicine trainees reported increased relevance of teaching content to their practice and exam preparation. Teaching session cancellations decreased notably, contributing to a more consistent learning environment. Importantly, the quality of teaching was rated more highly by trainees, reflecting the effectiveness of the implemented changes. Figure 1 shows net negative and net positive satisfaction for the 11 questions (see Materials & Methods) used to assess trainees’ teaching satisfaction. Five-point Likert scales were used for each question, with the neutral option being assigned a value of 0, the two negative options values of -1 and -2, and the two positive options assigned values of +1 and +2. These numerical values were added up across trainees, resulting in a score that reflected overall net satisfaction for that specific question. Hence, a score less than 0 reflects an overall negative response to a question and a score greater than 0 an overall positive response to a question.

In view of the small sample size and a non-representative sample, only descriptive statistics were used to summarize the findings. No statistical test was run to assess for statistical significance.

## Discussion

**Figure 2:**
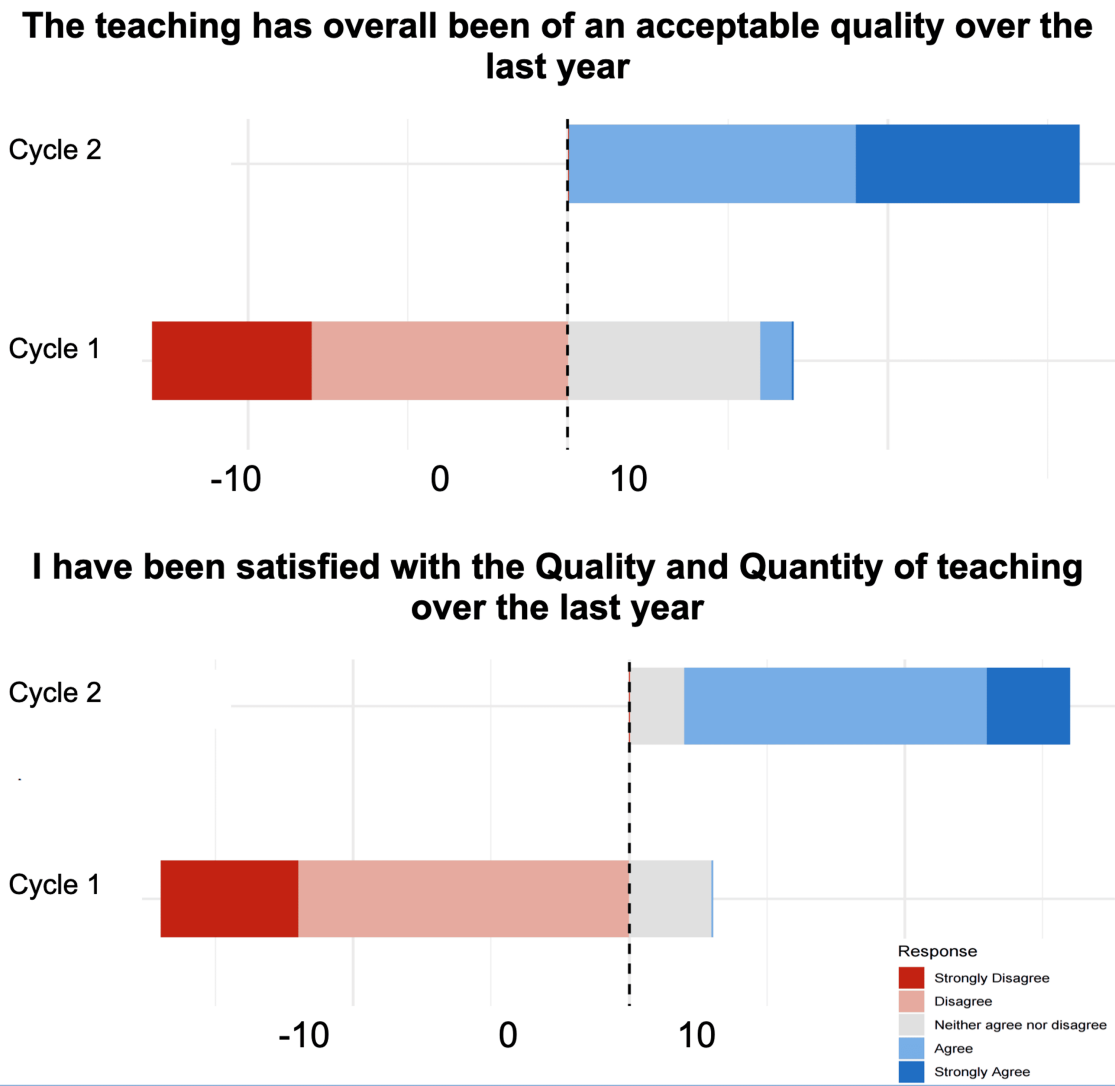
Overall satisfaction with quality and quantity of teaching across both cycles Five-point Likert scales were used for both questions, with the neutral option being assigned a value of 0, the two negative options values of -1 and -2, and the two positive options assigned values of +1 and +2. Values on the X-axis reflect summed total scores across trainees; hence, values greater than 0 reflect overall satisfaction and values below 0 overall dissatisfaction.

This quality improvement project showed that relatively simple, low-cost interventions have the potential to markedly improve internal medicine trainees’ perception of their teaching (Figure 2), which in turn has been associated with improved patient care [7]. Collecting feedback from trainees is crucial and has previously been associated with better outcomes [8]. Currently, the national healthcare system in the UK is being significantly affected by strikes of the trainee doctor workforce, which has the potential to impact patient care negatively [9]. Beyond more complex and expensive changes, such as improving doctors’ pay, interventions at a local level, for instance, those centered around trainee teaching, may have the potential to help ameliorate the workforce crisis affecting the British healthcare system. This quality improvement project demonstrates that incorporating trainees’ feedback, optimizing teaching formats, and promoting in-person interactions can enhance the quality and satisfaction of teaching during their internal medicine training.

Limitations

There are several important limitations to this quality improvement project. First, participants were not blinded to the intervention. They were informed of the purpose of the project (i.e., to improve their postgraduate teaching) and they were aware of the changes that had been made to their teaching program following the first cycle of data collection. Second, while 86% of internal medicine trainees at our hospital provided feedback, it was nevertheless a relatively small sample size with only 22 participants. It is therefore not necessarily generalizable to other hospitals and institutions. Third, this study specifically concerned internal medicine trainees and did not assess teaching for other specialties.

## Conclusions

In summary, this quality improvement project highlights the positive impact of simple, low-cost interventions on internal medicine trainees’ perceptions of teaching. By taking trainee feedback into account and changing the format of teaching accordingly, significant improvements in teaching relevance, reliability and overall quality were made. Of particular relevance were the inclusion of study leave in trainees’ rotas, the move from online to in-person teaching, and having longer dedicated teaching afternoons. This project shows the importance of adapting lessons to the feedback of trainees in order to improve their training experience. This, in turn, has the potential to positively impact the retention crisis among UK trainee doctors. We suggest that similar projects should be conducted in other medical specialties to assess for generalizability.
